# Supplementary material for: Thiodiketopiperazines Produced by Penicillium crustosum and Their Activities to Promote Gastrointestinal Motility
Source: Molecules. 2019 Jan 15;24(2):299. doi: 10.3390/molecules24020299 (PMC6359346; doi:10.3390/molecules24020299)
Supplement: Supplementary file 1 [file molecules-24-00299-s001.pdf]

Supporting information

Thio-diketopiperazines Produced by *Penicillium crustosum* and Their Activities to Promote Gastrointestinal Motility

Xin He§[a], Jing Yang§[b], Ling Qiu[a], Dan Feng[a], Feng Ju[a], Lu Tan[a], Yu-Zhi Li[a], Yu-Cheng Gu[c], Zhen Zhang[a], Da-Le Guo*[a] and Yun Deng*[a]

a. The Ministry of Education Key Laboratory of Standardization of Chinese Herbal Medicine, State Key Laboratory, Breeding Base of Systematic Research Development and Utilization of Chinese Medicine Resources, School of Pharmacy, Chengdu University of Traditional Chinese Medicine, Chengdu 611137, P R China

b. State Key Laboratory of Phytochemistry and Plant Resources in West China, Kunming Institute of Botany, Chinese Academy of Sciences, Kunming 650201, P R China

c. Syngenta Jealott's Hill International Research Centre, Berkshire RG42 6EY, UK

*Corresponding author. Tel/Fax: +86 028 61800232.

E-*mail address*: guodale@cdutcm.edu.cn (Dale Guo)

dengyun2000@hotmail.com (Yun Deng)

# Xin He and Jing Yang have contributed equally to this work

Tables of Contents

S1. HRESIMS spectrum of **1**

S2. ^{1}H NMR spectrum (400 MHz, acetone-d_6) of **1**

S3. ^{13}C NMR spectrum (100 MHz, acetone-d_6) of **1**

S4. ^{1}H-^{1}H COSY spectrum (acetone-d_6) of **1**

S5. HSQC spectrum (acetone-d_6) of **1**

S6. HMBC spectrum (acetone-d_6) of **1**

S7. NOESY spectrum (acetone-d_6) of **1**

S8. ^{1}H NMR spectrum (400 MHz, pyridine-d_5) of *S*-MTPA-**1**

S9. ^{1}H NMR spectrum (400 MHz, pyridine-d_5) of *R*-MTPA-**1**

S10. Experimental ECD spectrum (methanol) of **1**

S11. HRESIMS spectrum of **2**

S12. ^{1}H NMR spectrum (400 MHz, chloroform-*d*) of **2**

S13. ^{13}C NMR spectrum (100 MHz, chloroform-*d*) of **2**

S14. ^{1}H-^{1}H COSY spectrum (chloroform-*d*) of **2**

S15. HSQC spectrum (chloroform-*d*) of **2**

S16. HMBC spectrum (chloroform-*d*) of **2**

S17. NOESY spectrum (chloroform-*d*) of **2**

S18. ^{1}H NMR spectrum (400 MHz, pyridine-d_5) of *S*-MTPA-**2**

S19. ^{1}H NMR spectrum (400 MHz, pyridine-d_5) of *R*-MTPA-**2**

S20. Experimental ECD spectrum (methanol) of **2**

S21. HRESIMS spectrum of **3**

S22. ^{1}H NMR spectrum (400 MHz, chloroform-*d*) of **3**

S23. ^{13}C NMR spectrum (100 MHz, chloroform-*d*) of **3**

S24. ^{1}H-^{1}H COSY spectrum (chloroform-*d*) of **3**

S25. HSQC spectrum (chloroform-*d*) of **3**

S26. HMBC spectrum (chloroform-*d*) of **3**

S27. NOESY spectrum (chloroform-*d*) of **3**

S28. ^{1}H NMR spectrum (400 MHz, pyridine-d_5) of *S*-MTPA-**3**

S29. ^{1}H NMR spectrum (400 MHz, pyridine-d_5) of *R*-MTPA-**3**

S30. Experimental ECD spectrum (methanol) of **3**

S31. Effect of Compound **1-5** on the gastrointestinal motility of zebrafish

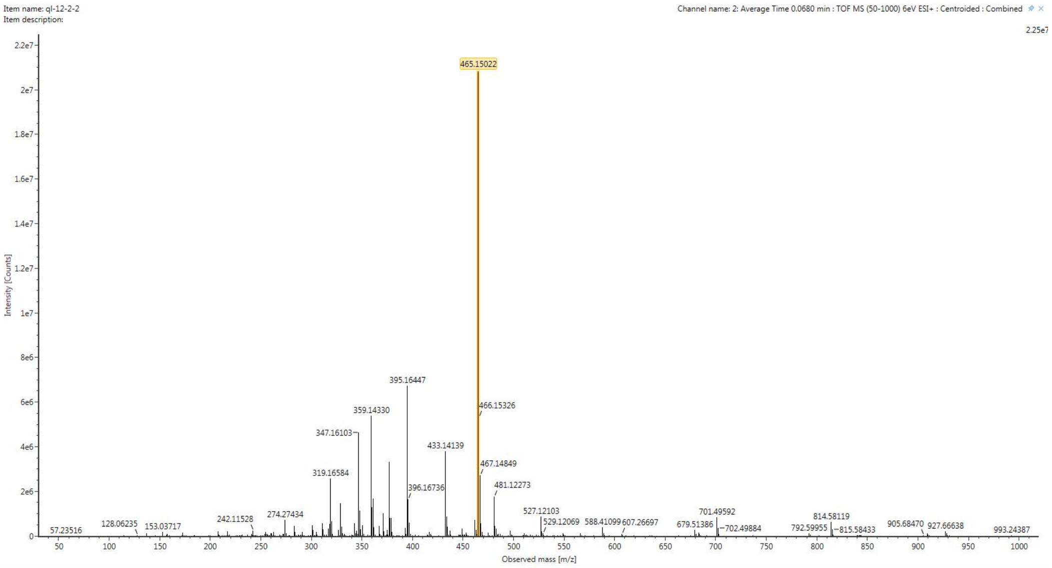

S1. HRESIMS spectrum of **1**

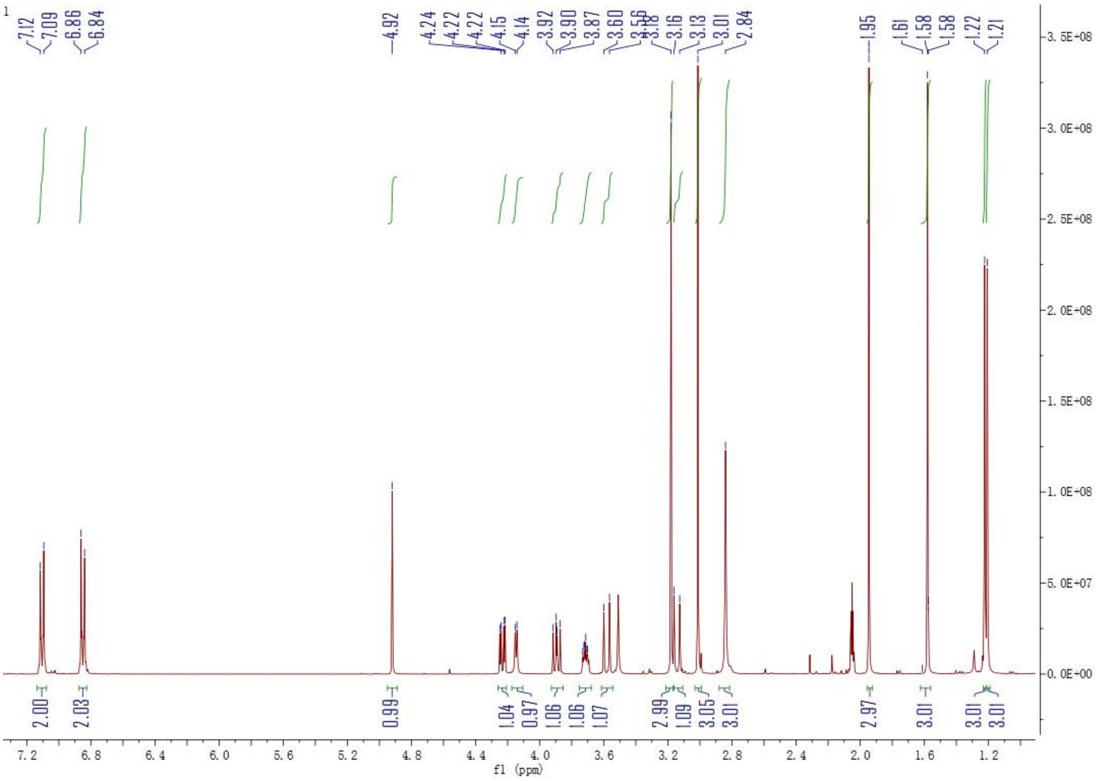

S2. ^{1}H NMR spectrum (400 MHz, acetone-d_6) of **1**

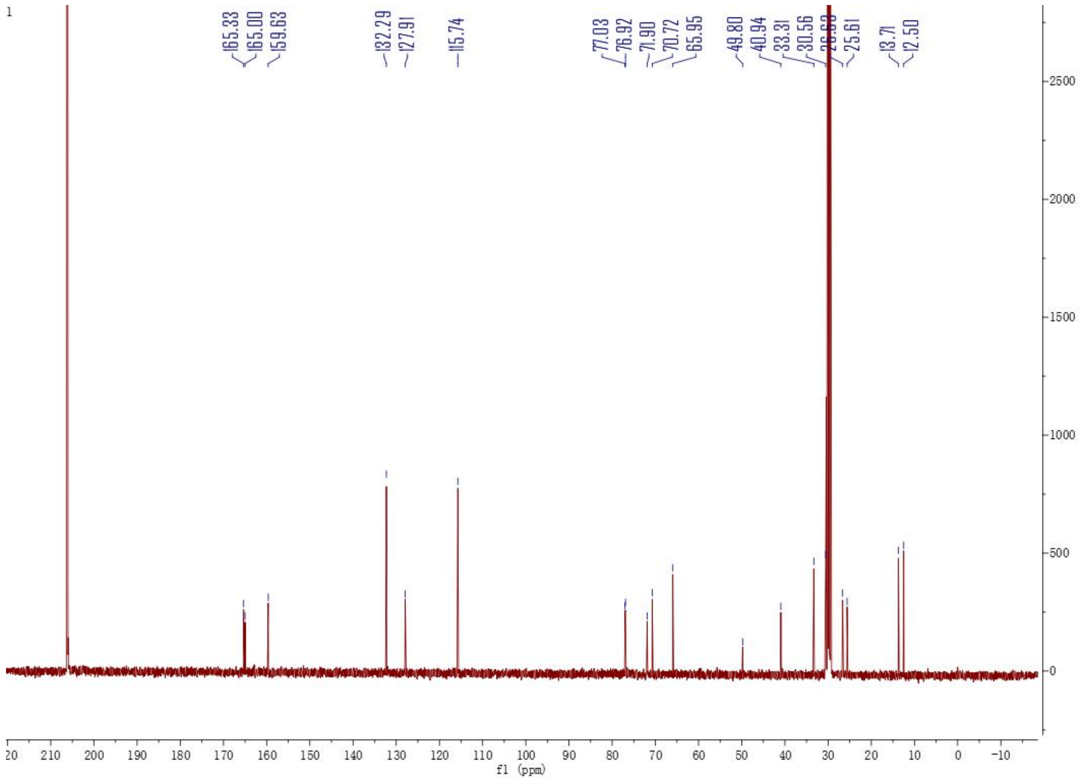

S3. ^{13}C NMR spectrum (100 MHz, acetone-d_6) of **1**

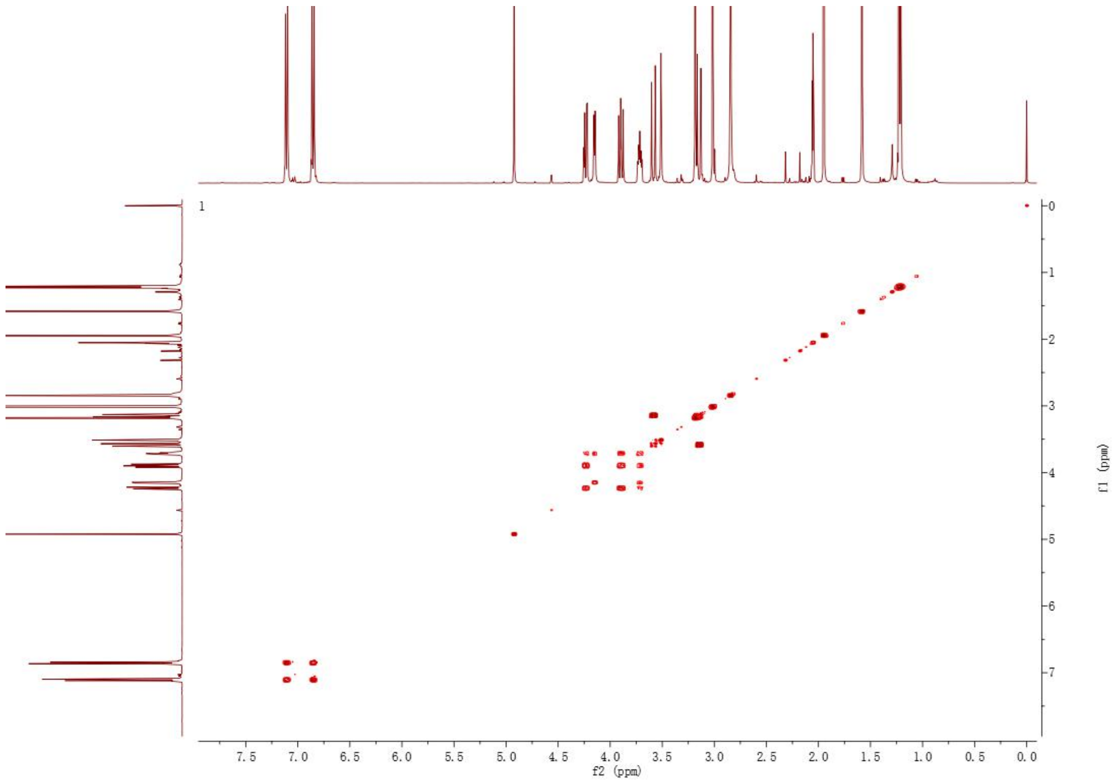

S4. ^{1}H-^{1}H COSY spectrum (acetone-d_6) of **1**

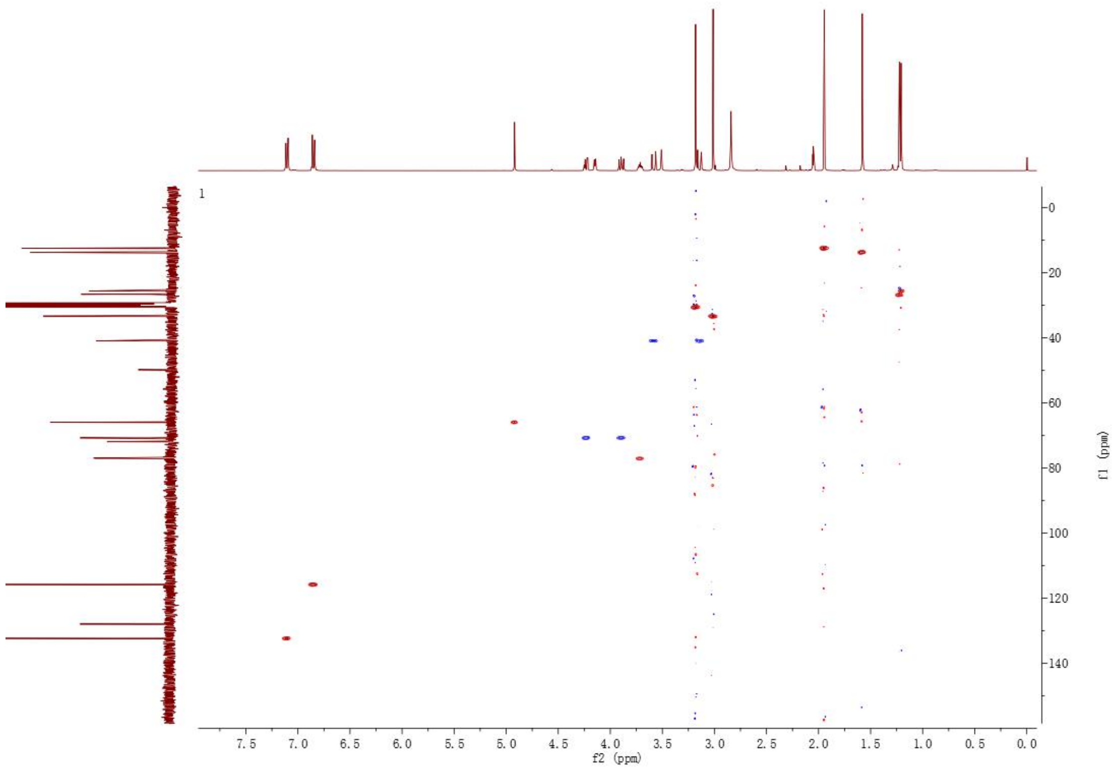

S5. HSQC spectrum (acetone-d_6) of **1**

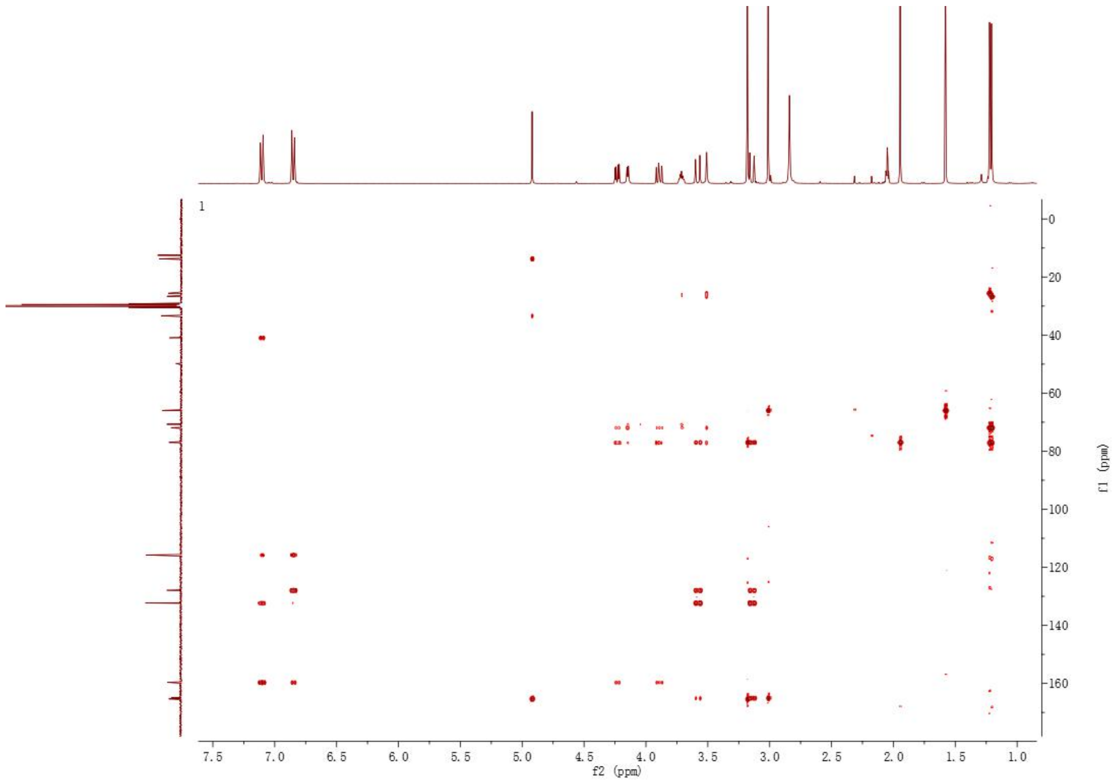

S6. HMBC spectrum (acetone-d_6) of **1**

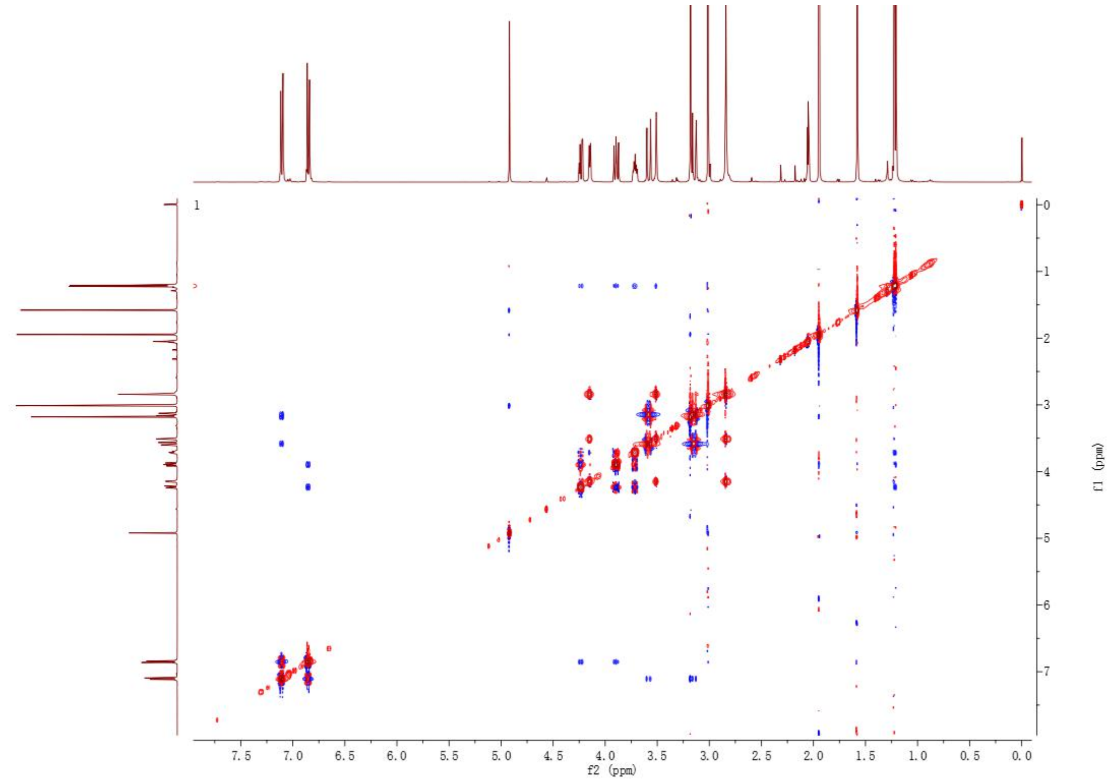

S7. NOESY spectrum (acetone-d_6) of **1**

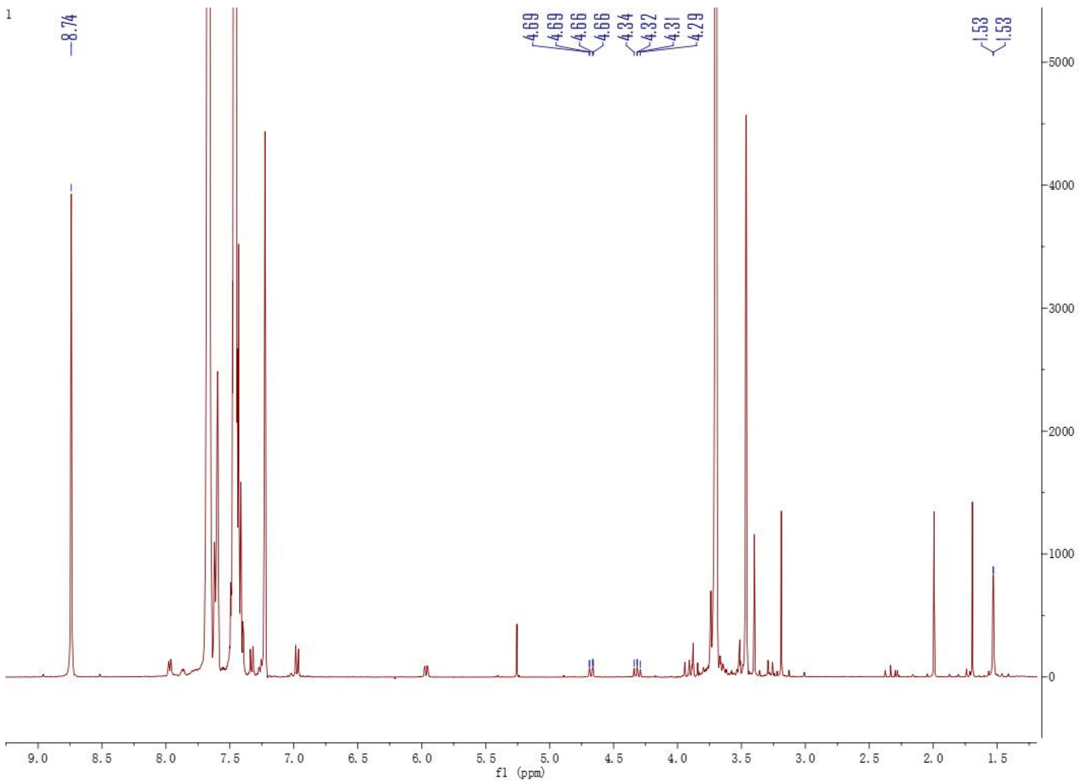

S8. ^{1}H NMR spectrum (400 MHz, pyridine-d_5) of *S*-MTPA-**1**

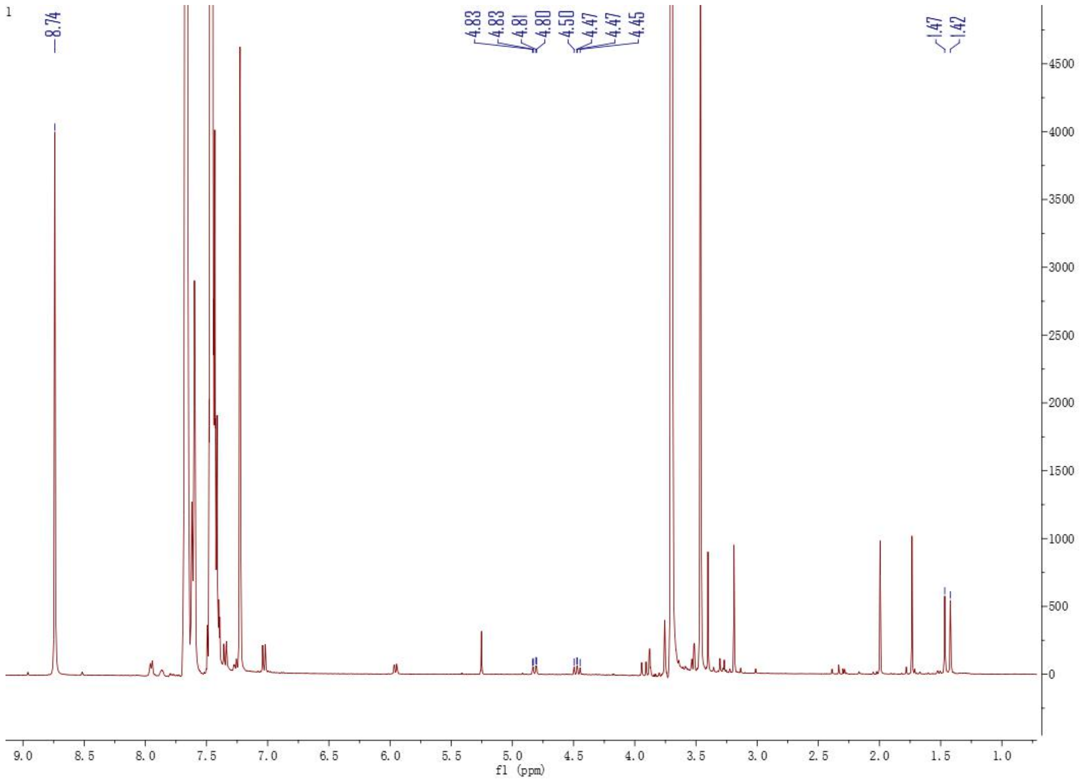

S9. ^{1}H NMR spectrum (400 MHz, pyridine-d_5) of *R*-MTPA-**1**

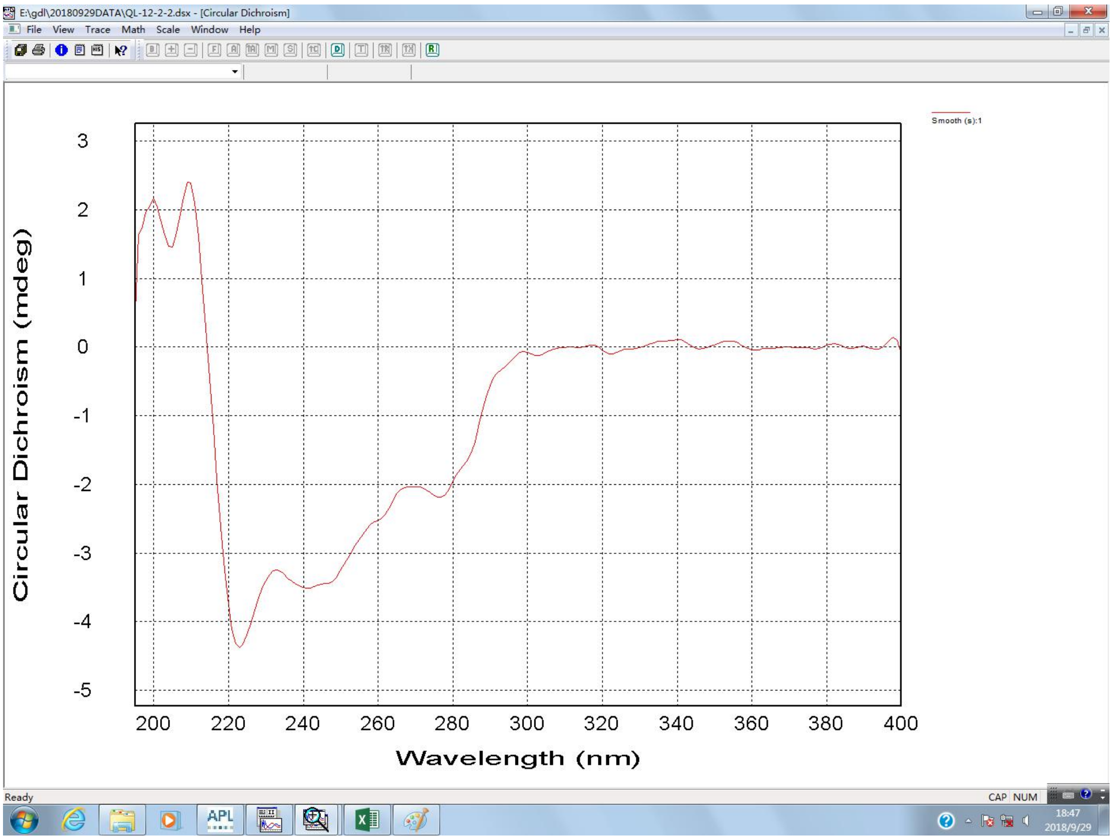

S10. Experimental ECD spectrum (methanol) of **1**

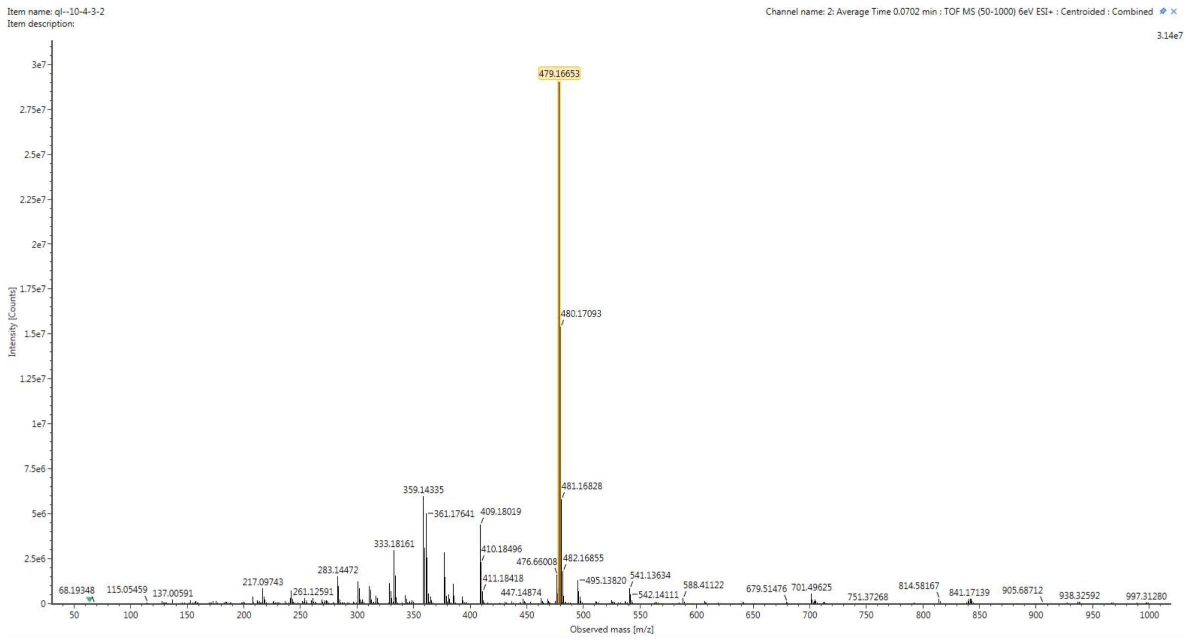

S11. HRESIMS spectrum of **2**

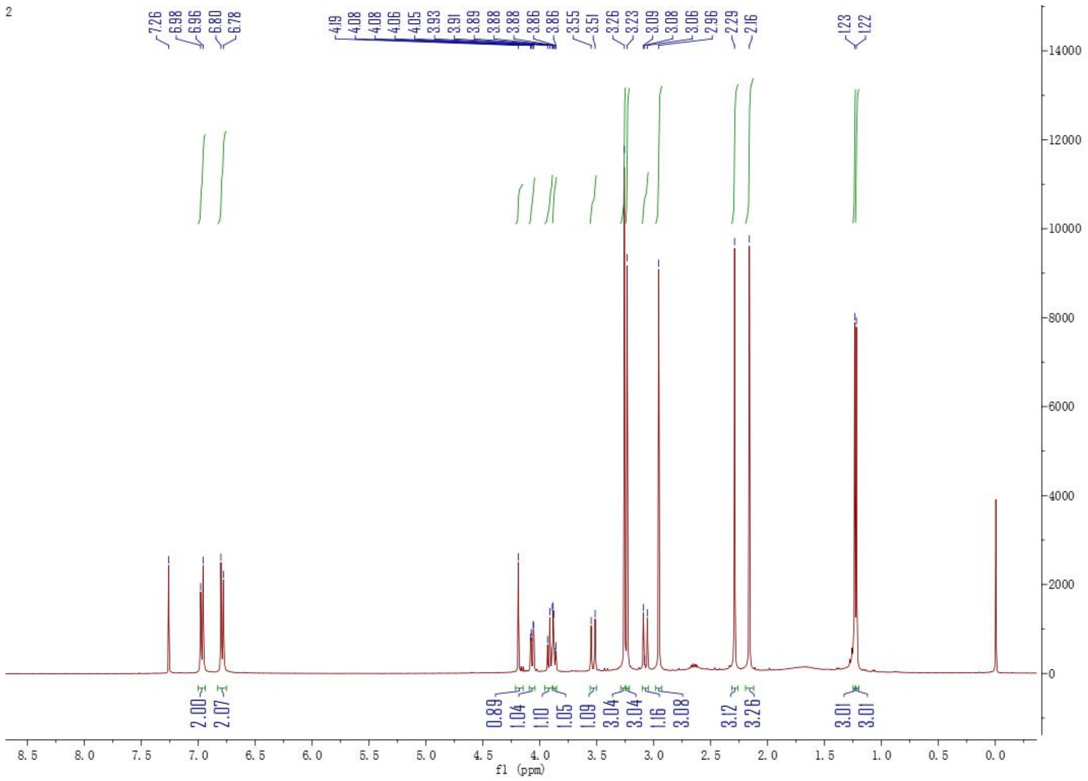

S12. ^{1}H NMR spectrum (400 MHz, chloroform-*d*) of **2**

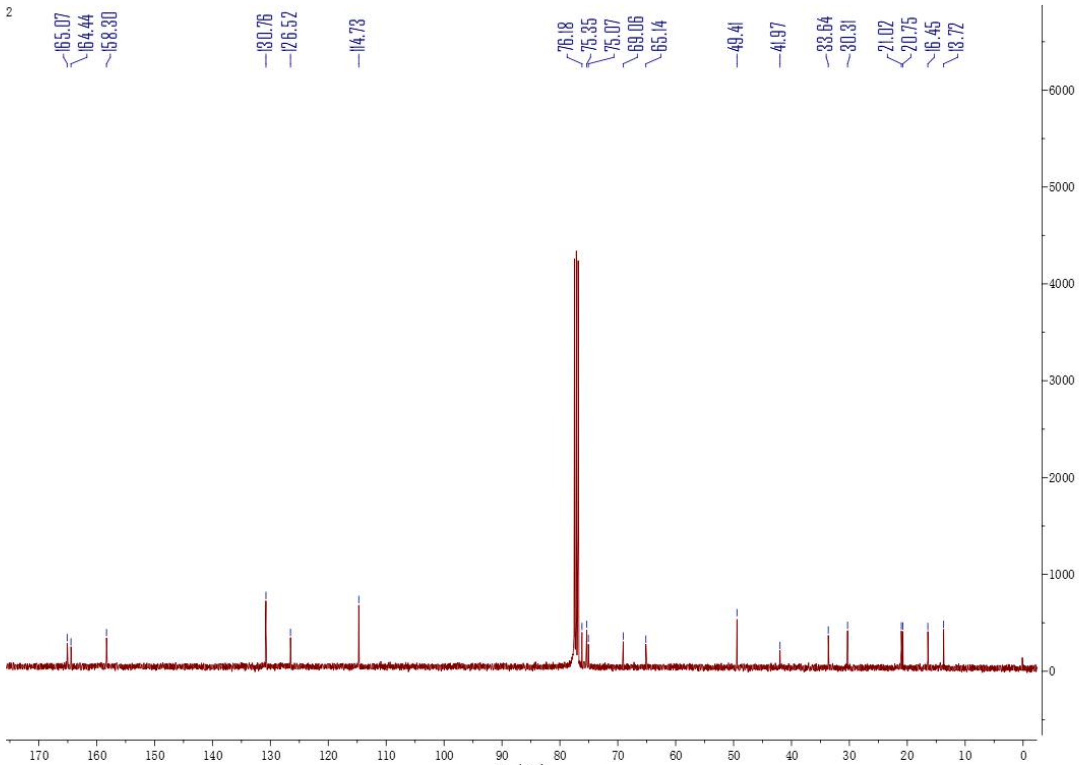

S13. ^{13}C NMR spectrum (100 MHz, chloroform-*d*) of **2**

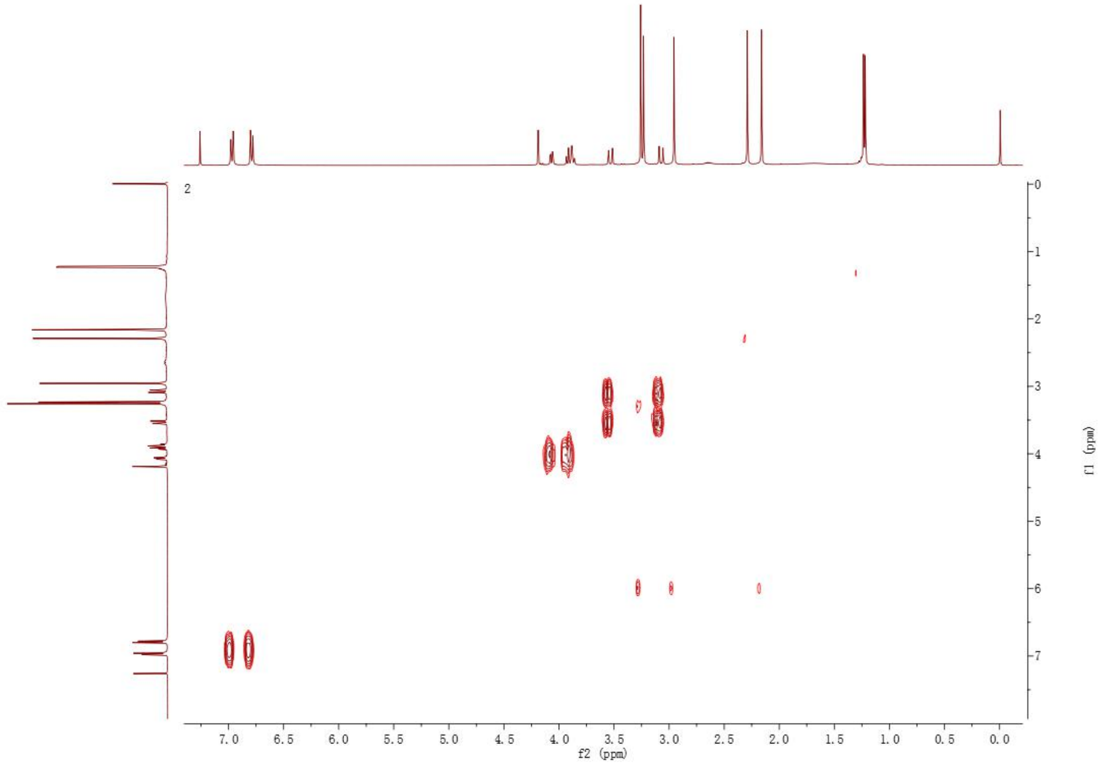

S14. ^{1}H-^{1}H COSY spectrum (chloroform-*d*) of **2**

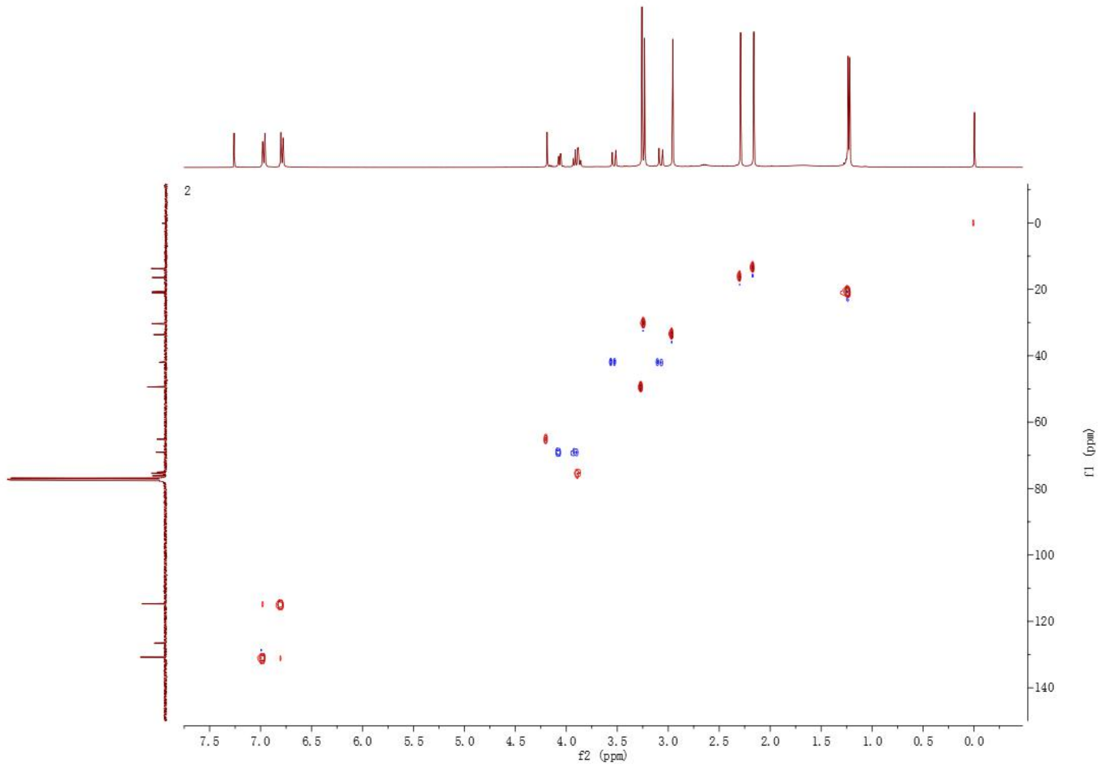

S15. HSQC spectrum (chloroform-*d*) of **2**

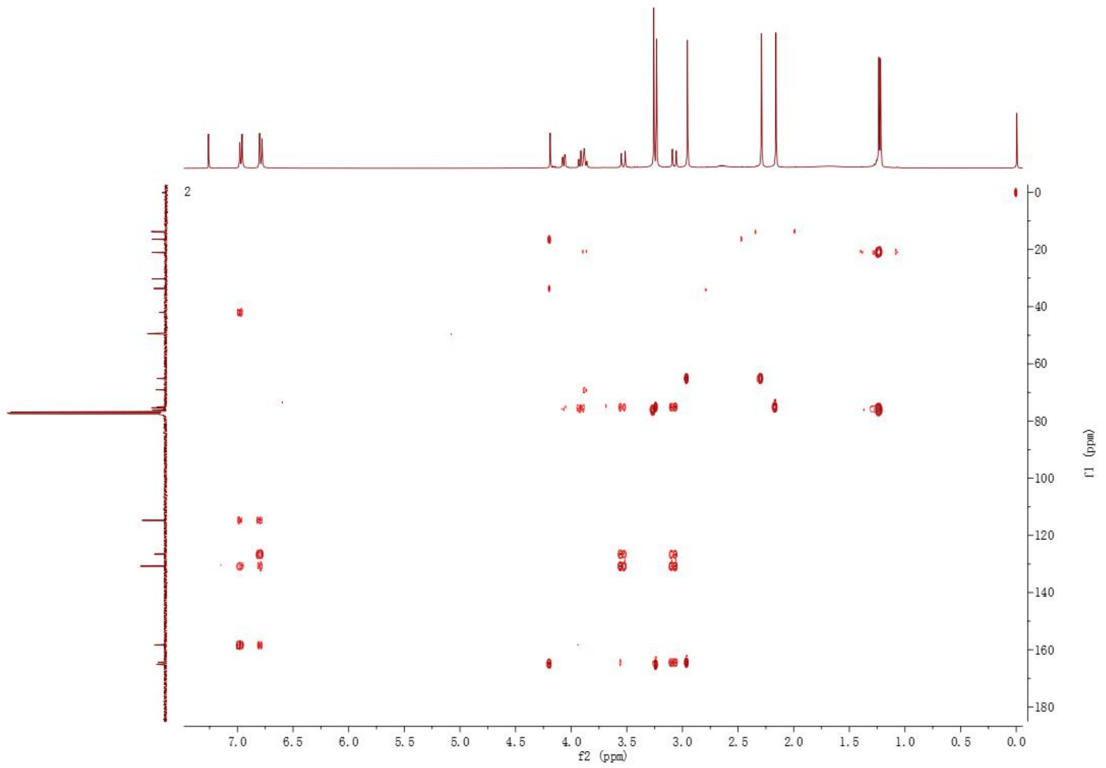

S16. HMBC spectrum (chloroform-*d*) of **2**

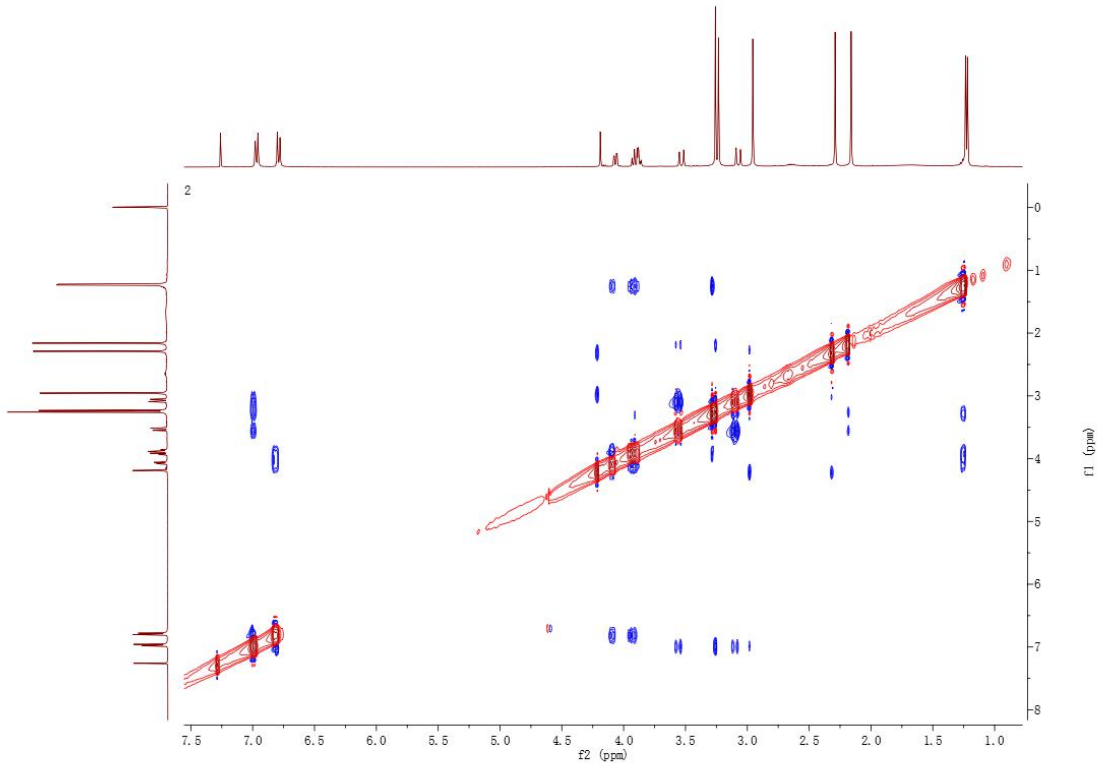

S17. NOESY spectrum (chloroform-*d*) of **2**

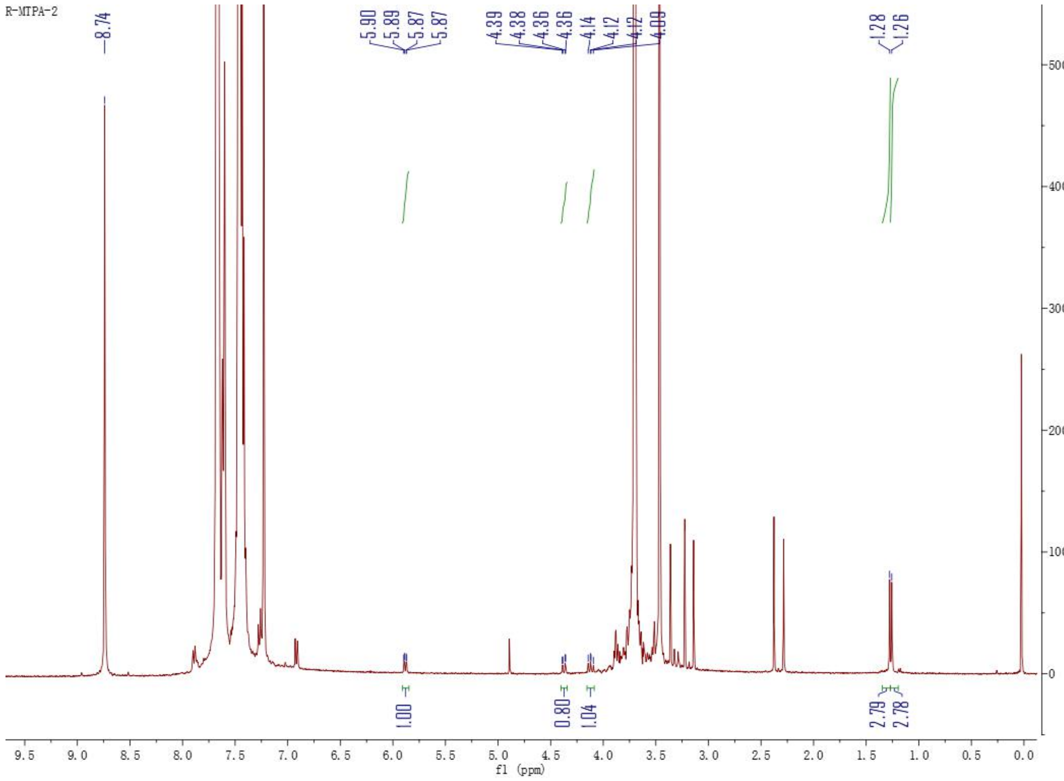

S18. ^{1}H NMR spectrum (400 MHz, pyridine-d_5) of *S*-MTPA-**2**

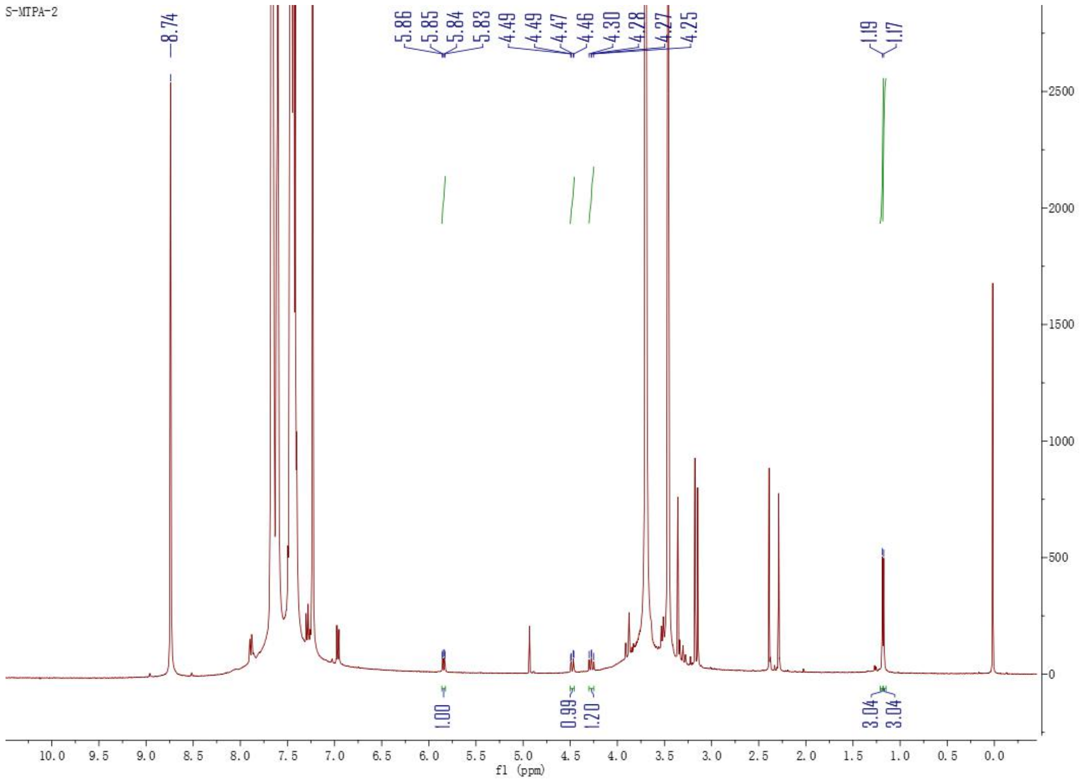

S19. ^{1}H NMR spectrum (400 MHz, pyridine-d_5) of *R*-MTPA-**2**

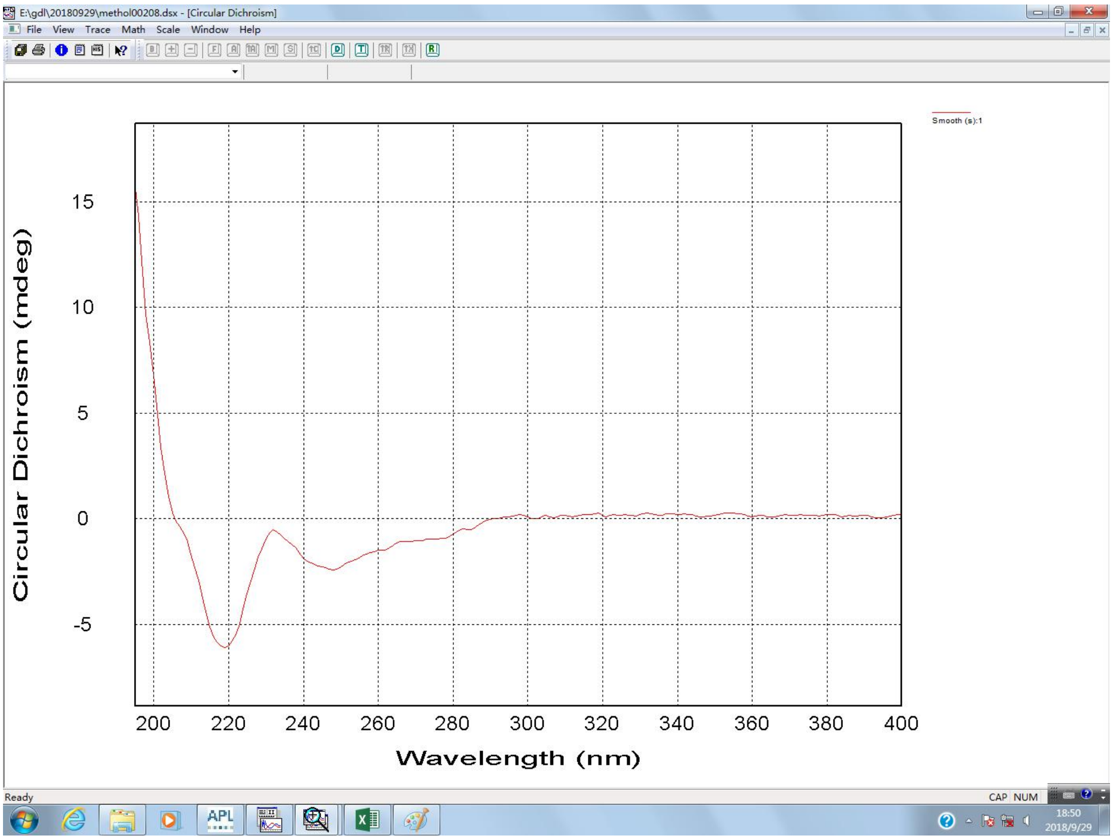

S20. Experimental ECD spectrum (methanol) of **2**

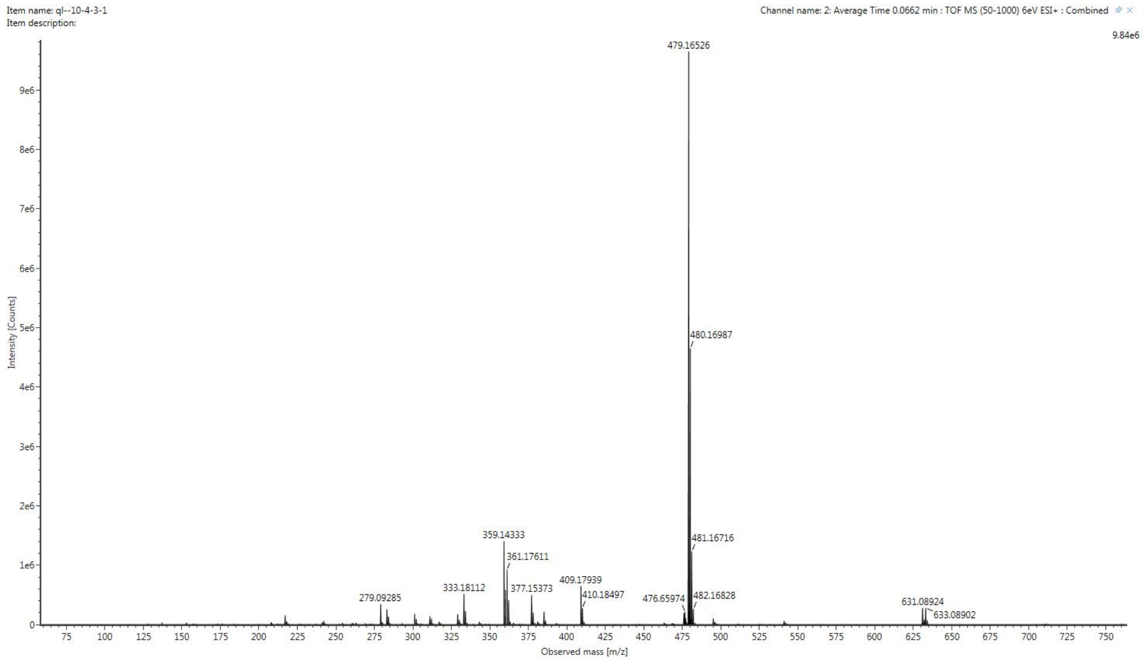

S21. HRESIMS spectrum of **3**

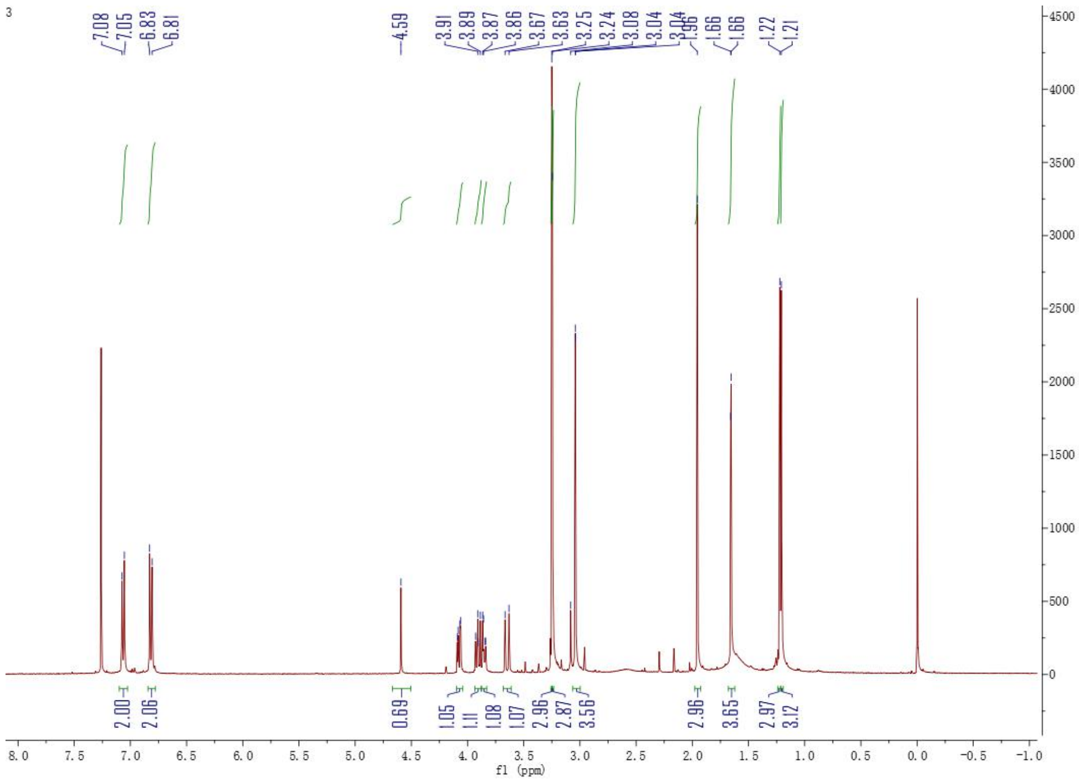

S22. ^{1}H NMR spectrum (400 MHz, chloroform-*d*) of **3**

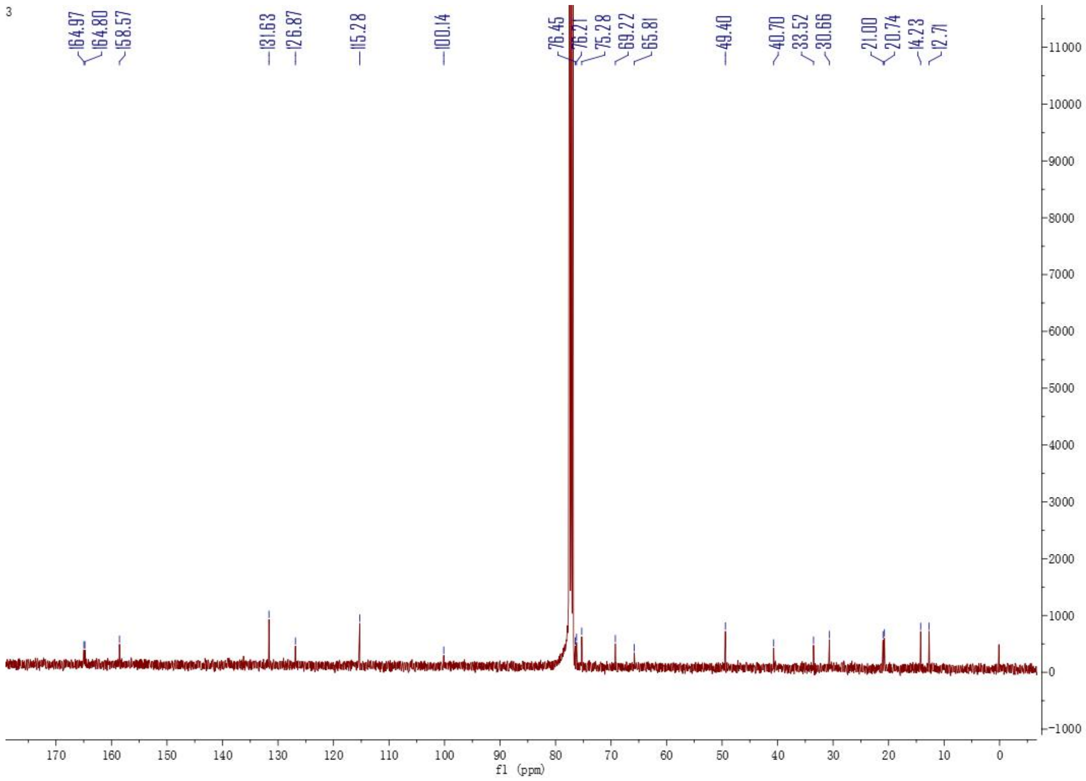

S23. ^{13}C NMR spectrum (100 MHz, chloroform-*d*) of **3**

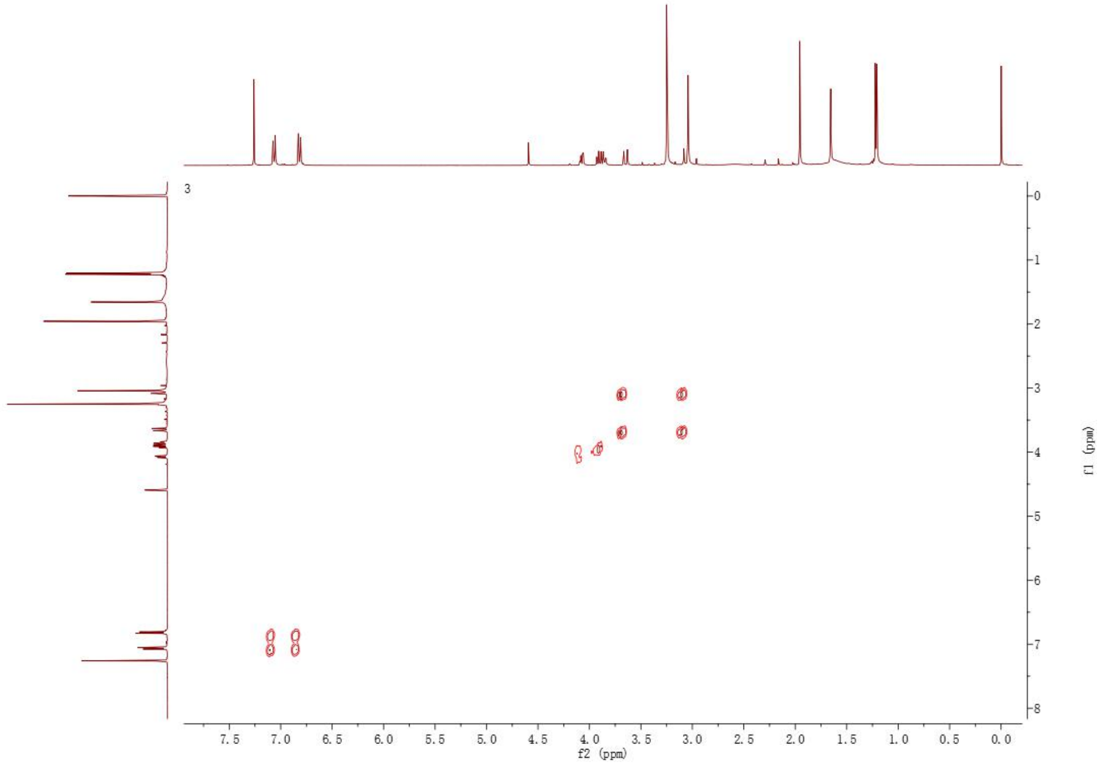

S24. ^{1}H-^{1}H COSY spectrum (chloroform-*d*) of **3**

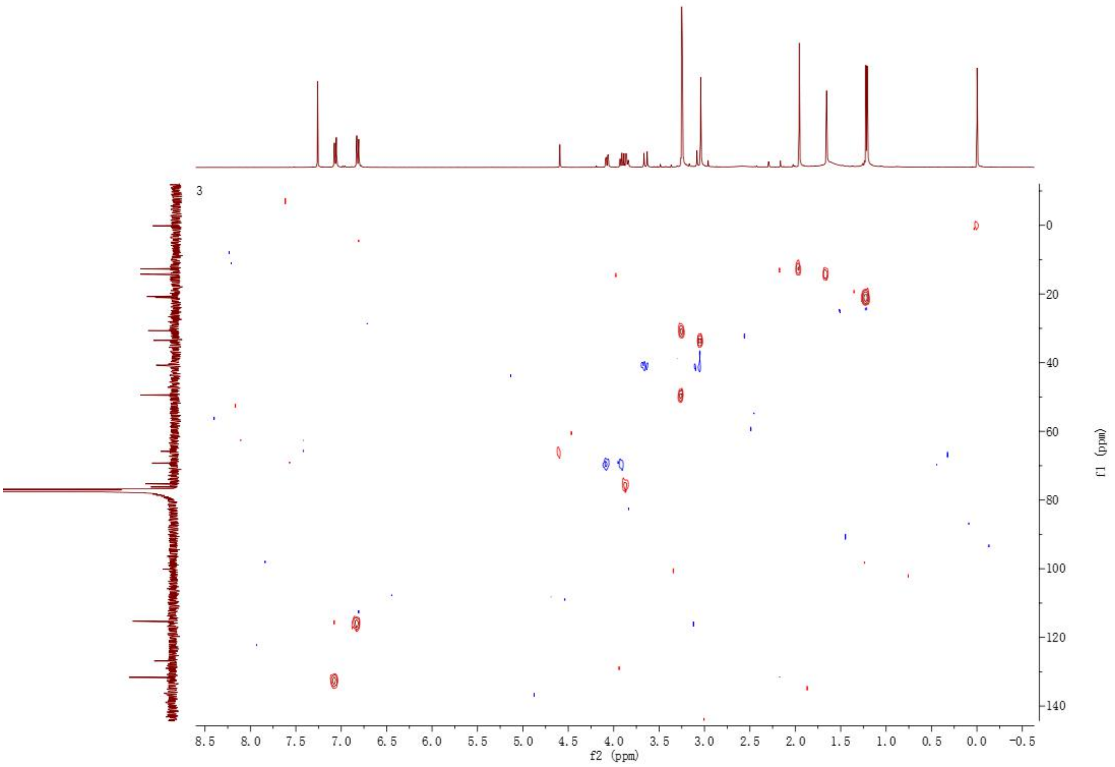

S25. HSQC spectrum (chloroform-*d*) of **3**

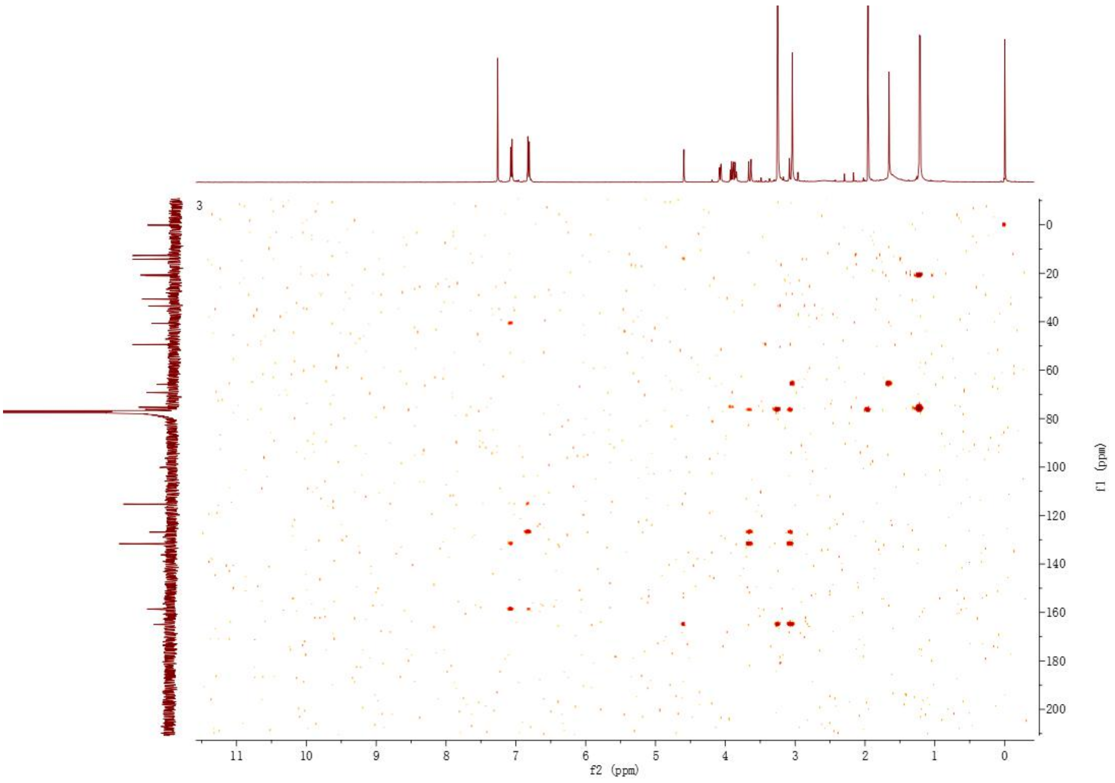

S26. HMBC spectrum (chloroform-*d*) of **3**

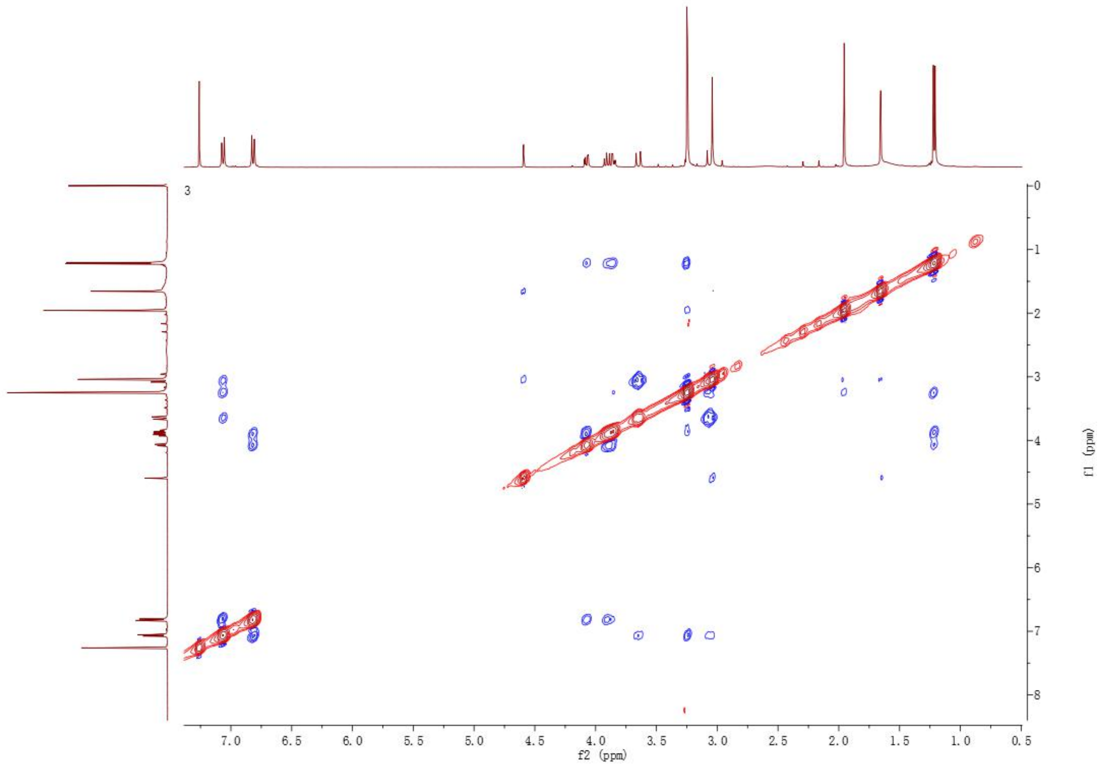

S27. NOESY spectrum (chloroform-*d*) of **3**

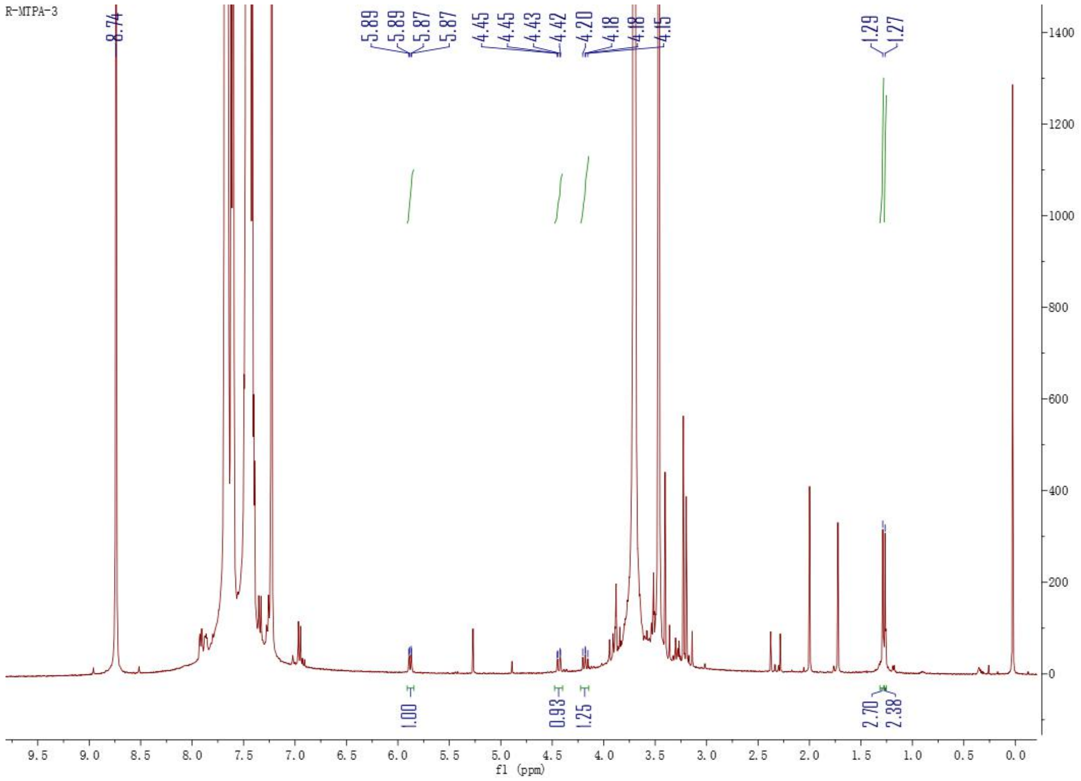

S28. ^{1}H NMR spectrum (400 MHz, pyridine-d_5) of *S*-MTPA-**3**

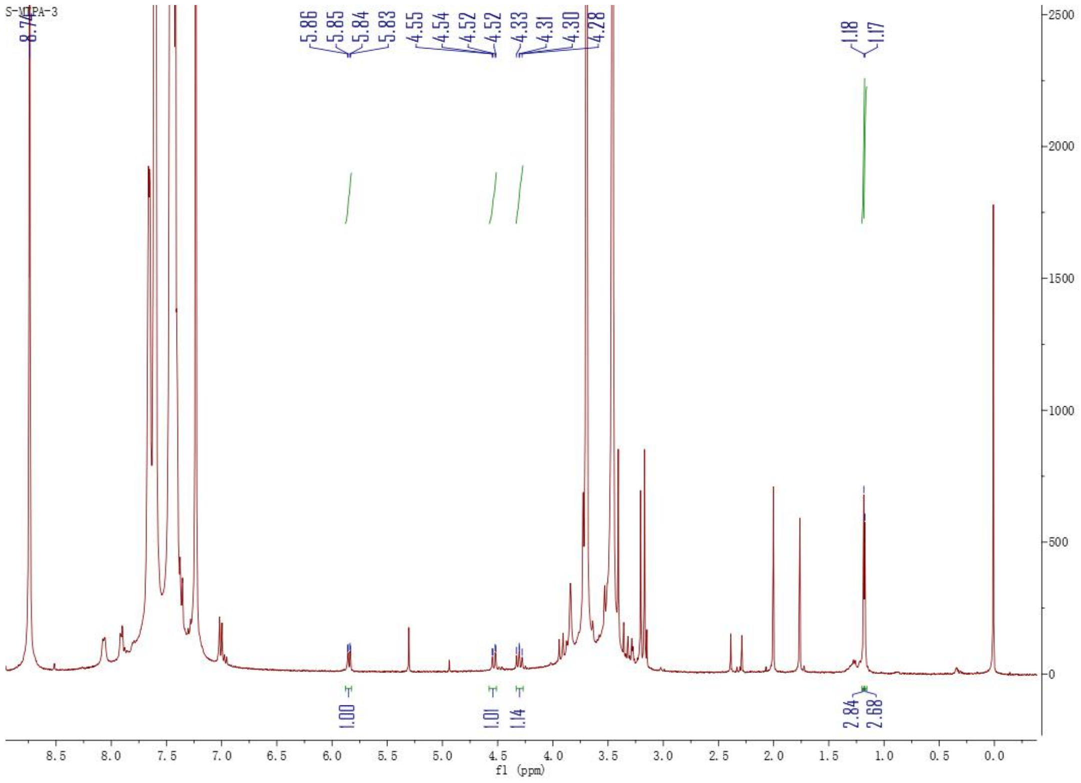

S29. ^{1}H NMR spectrum (400 MHz, pyridine-d_5) of *R*-MTPA-**3**

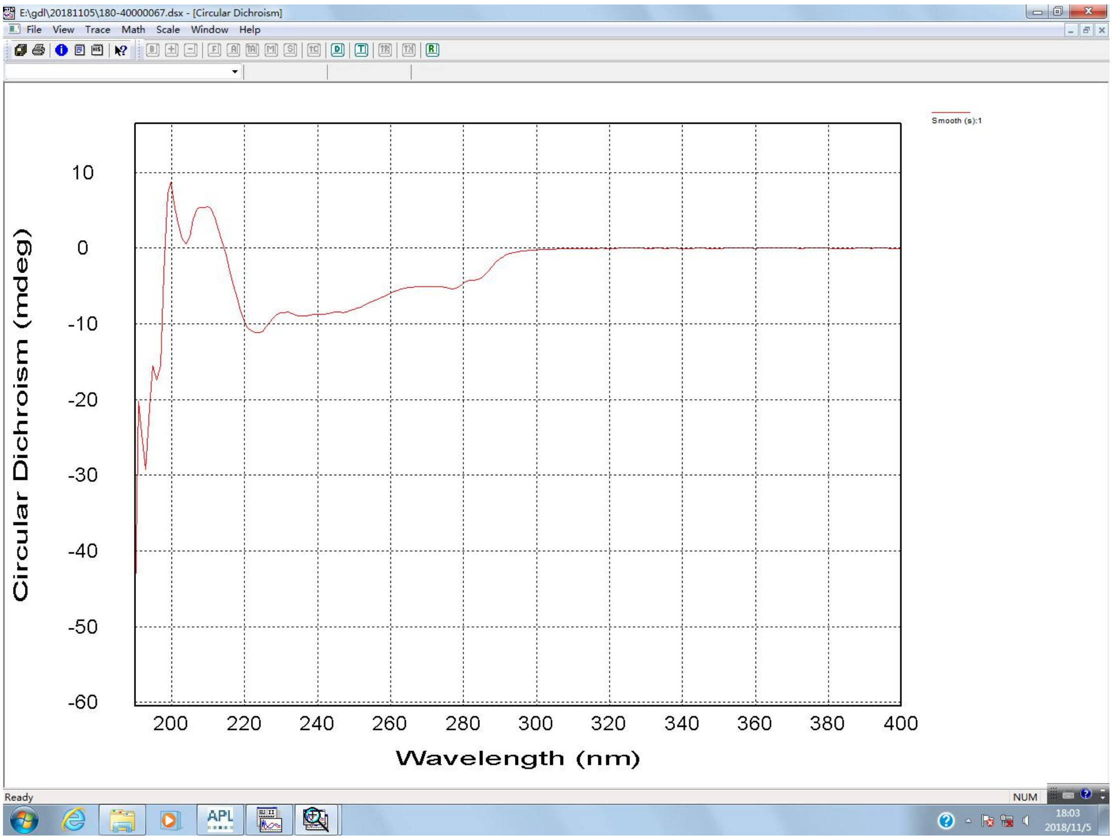

S30. Experimental ECD spectrum (methanol) of **3**

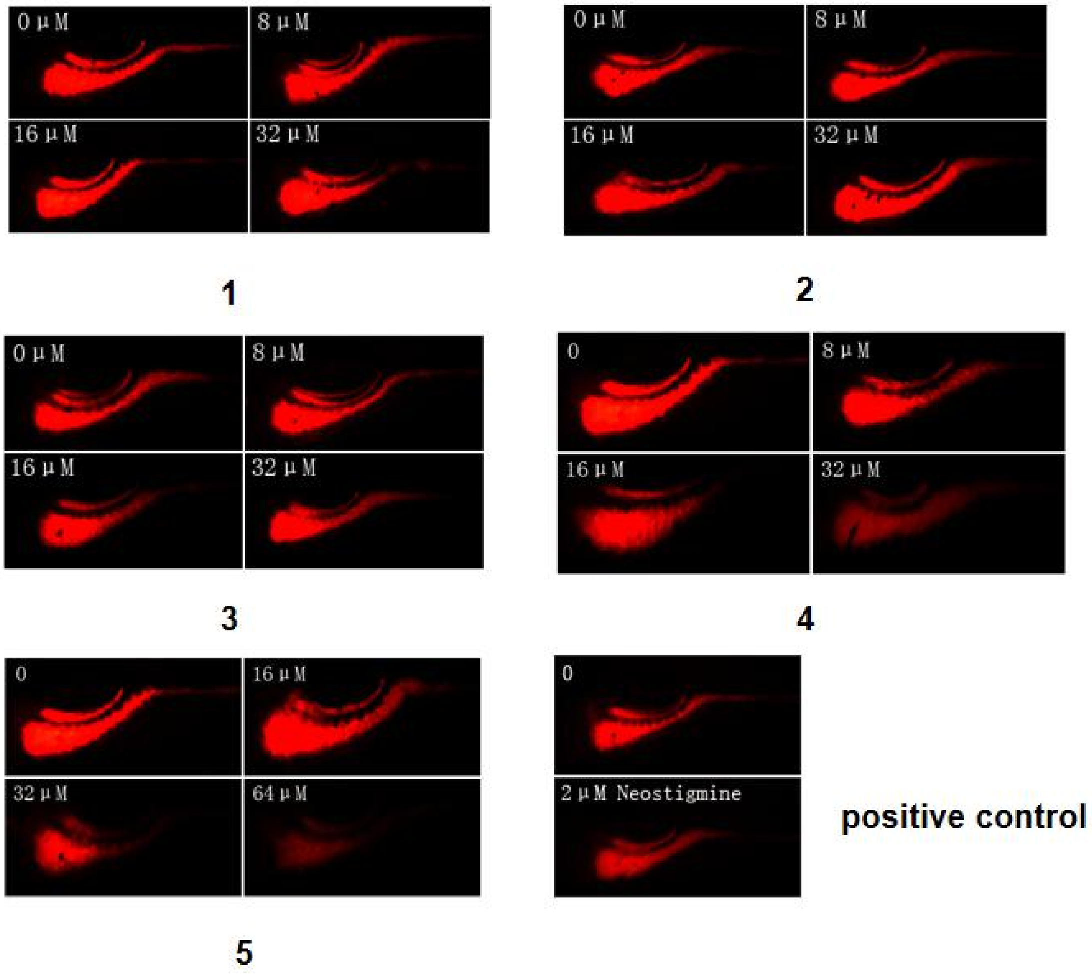

S31. Effect of Compound **1**-**5** on the gastrointestinal motility of zebrafish
